# Supplementary material for: HIVprotI: an integrated web based platform for prediction and design of HIV proteins inhibitors
Source: J Cheminform. 2018 Mar 9;10:12. doi: 10.1186/s13321-018-0266-y (PMC5845081; doi:10.1186/s13321-018-0266-y)
Supplement: Supplementary file 2 — Additional file 2. Source code of HIVProtI web server. [file 13321_2018_266_MOESM2_ESM.zip › HIVprotI_Source-code/hivprotI/weka-3-6-6/doc/weka/clusterers/forOPTICSAndDBScan/DataObjects/package-summary.html]

weka.clusterers.forOPTICSAndDBScan.DataObjects


---


|  |  |  |
| --- | --- | --- |
| **Overview** \| **Package** \| Class \| **Tree** \| **Deprecated** \| **Index** \| **Help** \| **Weka's home** | |  |
| **PREV PACKAGE**   **NEXT PACKAGE** | **FRAMES**    **NO FRAMES**     **All Classes** |  |


---

## Package weka.clusterers.forOPTICSAndDBScan.DataObjects

| **Interface Summary** | |
| --- | --- |
| **DataObject** | DataObject.java   Authors: Rainer Holzmann, Zhanna Melnikova-Albrecht, Matthias Schubert   Date: Aug 19, 2004   Time: 5:48:59 PM   $ Revision 1.4 $ |

| **Class Summary** | |
| --- | --- |
| **EuclidianDataObject** | EuclidianDataObject.java   Authors: Rainer Holzmann, Zhanna Melnikova-Albrecht, Matthias Schubert   Date: Aug 19, 2004   Time: 5:50:22 PM   $ Revision 1.4 $ |
| **ManhattanDataObject** | ManhattanDataObject.java   Authors: Rainer Holzmann, Zhanna Melnikova-Albrecht, Matthias Schubert   Date: Aug 19, 2004   Time: 5:50:22 PM   $ Revision 1.4 $ |

---


|  |  |  |
| --- | --- | --- |
| **Overview** \| **Package** \| Class \| **Tree** \| **Deprecated** \| **Index** \| **Help** \| **Weka's home** | |  |
| **PREV PACKAGE**   **NEXT PACKAGE** | **FRAMES**    **NO FRAMES**     **All Classes** |  |


---
